# *Yersinia* Virulence Factor YopM Induces Sustained RSK Activation by Interfering with Dephosphorylation

**DOI:** 10.1371/journal.pone.0013165

**Published:** 2010-10-05

**Authors:** Moritz Hentschke, Laura Berneking, Cristina Belmar Campos, Friedrich Buck, Klaus Ruckdeschel, Martin Aepfelbacher

**Affiliations:** 1 Institute of Medical Microbiology, Virology and Hygiene, University Medical Center Eppendorf, Hamburg, Germany; 2 Institute of Clinical Chemistry; University Medical Center Eppendorf, Hamburg, Germany; Technical University Munich, Germany

## Abstract

**Background:**

Pathogenic yersiniae inject several effector proteins (Yops) into host cells, which subverts immune functions and enables the bacteria to survive within the host organism. YopM, whose deletion in enteropathogenic yersiniae results in a dramatic loss of virulence, has previously been shown to form a complex with and activate the multifunctional kinases PKN2 and RSK1 in transfected cells.

**Methodology/Principal Findings:**

In a near physiological approach with double-affinity-tagged YopM being translocated into the macrophage cell line J774A.1 via the natural type three secretion system of *Yersinia* we verified the interaction of YopM with PKN2 and RSK1 and detected association with additional PKN and RSK isoforms. In transfected and infected cells YopM induced sustained phosphorylation of RSK at its activation sites serine-380 and serine-221 even in the absence of signalling from its upstream kinase ERK1/2, suggesting inhibition of dephosphorylation. ATP-depletion and in vitro assays using purified components directly confirmed that YopM shields RSK isoforms from phosphatase activity towards serines 380 and 221.

**Conclusions/Significance:**

Our study suggests that during *Yersinia* infection YopM induces sustained activation of RSK by blocking dephosphorylation of its activatory phosphorylation sites. This may represent a novel mode of action of a bacterial virulence factor.

## Introduction

Yersiniae are gram-negative bacteria which belong to the family of the Enterobacteriaceae. The genus *Yersinia* comprises *Yersinia pestis*, the causative agent of plague, and *Yersinia enterocolitica* and *Yersinia pseudotuberculosis*, both of which cause gastrointestinal disease. Like many other gram negative bacteria yersiniae utilize a so called type III-secretion system (TTSS) to inject proteins into host cells [1]. The target cells are mostly cells of the immune system like phagocytes, macrophages, polymorphonuclear neutrophils, lymphocytes and dendritic cells [2], [3]. The injected virulence proteins (“*Yersinia* outer proteins”, Yops) subvert the immune cells, which permits extracellular persistence and proliferation of *Yersinia*. Six different Yops, YopE, YopT, YopO/YpkA, YopH, YopP/YopJ and YopM, are translocated and attack diverse aspects of cellular immunity which disturbs immune cell function at different levels [4], [5]. YopE, YopT and YopO/YopkA modulate small GTP-binding proteins of the Rho-family, which take part in the regulation of the actin cytoskeleton. YopH is a tyrosine phosphatase and dephosphorylates focal adhesion complexes, thereby destroying local connections between the cytoskeleton and the plasma membrane. The activities of YopH, YopE, YopT and YopO reorganize the actin cytoskeleton and prevent phagocytosis, which is essential for the uptake and degradation of bacteria by phagocytes. The effector protein YopP targets other cellular responses and inhibits the MAP-Kinases ERK, JNK and p38 and the activation of the transcription factor NFkappaB [6], [7]. YopP acetylates several MAP-kinase-kinases (MAPKKs) and inhibitor of kappaB kinase beta (IKKbeta) on critical residues resulting in impaired activation and hence disruption of the particular signalling pathways [8], [9]. This results in reduced expression of cytokines and apoptosis in macrophages.

In contrast, the intracellular actions of YopM remained largely obscure. Nevertheless, YopM has an important role for *in vivo* virulence of yersiniae. Deletion of the *yopM* gene in pathogenic yersiniae results in a dramatic loss of virulence [10]. The size of YopM differs between different strains and serotypes ranging from 42–54 kDa due to a variable number [11] and composition [12] of leucine-rich-repeats (LRRs) of which YopM is mostly comprised. In crystallization experiments YopM assembled into tetramers which formed hollow cylinders [12]. Early experiments suggested that YopM might be secreted into the extracellular space, where it was thought to associate with and inhibit thrombin [13]. Subsequent studies clearly showed that YopM is injected into the host cells together with the other Yops suggesting intracellular target molecules [14], yet a recent study found YopM to associate with the extracellular alpha1-antitrypsin, albeit with unknown consequences [15]. Intracellularly, YopM seems at least partly to be localized in the nucleus [16], [17], [18] and one study using microarray analysis found the dysregulation of several genes implicated in cellular growth and cell cycle control [19]. In contrast a second similarly designed study did not identify genes regulated by YopM [20]. Thus, it is currently unclear, whether YopM exerts a transcriptional effect on single genes. One study found a YopM dependent depletion of NK-cells during infection with pathogenic yersiniae, but the underlying mechanism remained elusive [21]. A more recent study by the same group gave a more complex picture with NK cell depletion seen only in the spleen but not in the liver and without significance during *Yersinia* infection [22]. Instead, Gr1+ polymorphonuclear neutrophils were suggested to be important for YopM mediated virulence.

First insights into the cellular actions of YopM came from a study by McDonald et al., who co-immunoprecipitated the kinases RSK1 and PKN2 with YopM from transfected cells [23]. YopM bound both kinases simultaneously assembling a trimeric complex. In this complex RSK1 and PKN2 were both shown to be activated by the presence of YopM as demonstrated by kinase assays with precipitated kinases. RSK1 was directly activated by YopM while PKN2 seemed to be activated subsequently by RSK1 in the complex. Yet, the underlying molecular mechanisms of this activation cascade were not further analysed. Although both kinases are involved in multiple cellular processes a direct link to a function in the immune system is lacking so far. The RSK family is constituted of four different isoforms (RSK1–4) which are activated by the ERK-signalling pathway through a complex cascade of consecutive phosphorylations of the RSK molecule. Phosphorylation of serine 573 (amino acid numbering refers to human RSK1 throughout) by ERK1/2 results in autophosphorylation at serine 380, which creates a docking site for another kinase, PDK1. PDK1 then phosphorylates serine 221 in the N-terminal kinase domain which ultimately targets substrates [24], [25]. The phosphorylation of serine 359/363 by ERK1/2 and additional yet unidentified kinases is also necessary for full activation [26]. RSK proteins have an important role in the “immediate early gene” transcription following stimulation of the cell by phosphorylating transcription factors like SRF and CREB. Other nuclear target proteins are c-fos, Nurr77, CBP, ATF4, p300 and several others. Cytosolic substrates include among others the sodium/proton exchanger NHE1, the proapoptotic protein BAD and the Glycogen synthetase kinase 3 (GSK3). RSK members have been implicated in regulating cell cycle control, phagocytosis, apoptosis and translation. A recent study also found RanBP3 to be a substrate of RSK, which may point towards a role in nucleocytoplasmic transport regulation [27]. Furthermore, RSK was identified as a central regulator of cellular mobility [28], [29]. The *in vivo* function of the individual RSK-isoforms is not well established. Inactivating mutations in the RSK2 gene in humans cause the Coffin-Lowry syndrome [30], yet Rsk2 knockout mice show only very moderate phenotypes [31]. Phenotypes of other RSK-knockout mice have not yet been published. PKN proteins are also multifunctional proteins [32]. Three different isoforms of PKN (PKN1-3) exist and like RSK these proteins have been found to be involved in multiple cellular processes. PKN proteins are regulated through their interaction with Rho-GTPases, PDK1 and various lipids. One current model of the activation of PKN assumes that active Rho-GTPases associate with PKN which provides a docking site for PDK1 [33]. After binding, PDK1 phosphorylates PKN at a specific threonine in the activation loop (T774 in PKN1 and T816 in PKN2). PKN2 participates in the regulation of the actin cytoskeleton, cell adhesion, and cell cycle progression [34]. Furthermore, PKN is involved in transcriptional regulation by phosphorylating transcription factors like the Androgenreceptor (AR), Estrogenreceptor (ER), Heat Shock Factor 1 (HSF1) and Histone H3 [35]. Phenotypes of knockout mice for the PKNs have not been published, yet and like for the RSK isoforms it is currently unknown whether functional redundancy exists between the isoforms.

Using physiological infection conditions, our present study confirms an earlier report that YopM forms a complex with members of the RSK and PKN kinase families. We found that YopM induces long lasting activation of RSK even in the absence of upstream ERK-signalling. In vivo and in vitro evidence is provided that YopM interferes with dephosphorylation of RSK most likely by shielding it from the action of phosphatases. Hence, we can provide a novel mode of action of a bacterial effector protein explaining how hyperphosphorylation and overactivation of RSKfamily kinases is achieved.

## Materials and Methods

### Antibodies and plasmids

#### Antibodies

Anti-flag was from Sigma and used in the dilution 1∶3000, anti-myc clone 9E10 was from Cell Signaling and used in the dilution 1∶1000, anti-HA high affinity was from Roche and used in the dilution 1∶1000, anti-phospho-S380RSK was from Cell signaling and used in the dilution 1∶1000, anti-phospho-S221RSK was from R&D systems and used in the dilution 1∶2000, anti-RSK1 was from Santa Cruz Bitotechnology and used in the dilution 1∶1000, anti-ERK1/2 was from Cell Signaling and used in the dilution 1∶1000, anti phospho-ERK1/2 was from Cell Signaling and used in the dilution 1∶1000, anti-YopM was a gift from Jürgen Heesemann and used in the dilution 1∶3000. Anti-actin was from Millipore and used in a dilution of 1∶1000. Secondary antibodies against rabbit-IgG, rat-IgG and mouse-IgG were all from GE Healthcare and Horse radish peroxidase conjugated. All used in a dilution of 1∶10000.

#### Plasmids

The following expression plasmids have been described previously: HA-tagged RSK1 [36](Addgene plasmid 13841), Flag-tagged PKN1 [37], Flag-tagged PKN2 [38], YopM (nucleotide sequence corresponding to the *Yersinia enterocolitica* WA Serotype O:8 YopM gene of the virulence plasmid pYV127/90; gi: 28372983) in pACYC184 [39]. Clones for in vitro translation have been obtained from ImaGenes GmbH (Berlin, Germany), except PKN1 for which the above mentioned expression clone was used: RSK1 (gi:15929012), RSK2 (gi:109730041), RSK3 (gi:27696716), RSK4 (gi:32450548), PKN2 (gi:30354737), PKN3 (gi:38565959). HA-tagged RSK2 was created by amplifying a RSK2 fragment from gi:109730041 (see above, RSK2 in pCR-BluntII-Topo) with AGGTACCGCTAGCCATGGCATACCCATACGACGTCCCAGACTACATGCCGCTGGCGCAGCTGGCGGA and TCTACCAAGATATCACGTTCCA: This fragment was digested with *Kpn*I and *Eco*RV and cloned into the similarly digested gi:109730041. From there the whole construct was cut out with *Nhe*I and *Not*I and cloned into the similarly digested vector pCI-neo. Myc-YopM was created by amplifying YopM from YopM in pACYC184 mentioned above with the primers Forward CTGACCATGGAATATGGTTTTGTTTGCAATGAA and Reverse GTCACTCGAGCTACTCAAAAACATCATCTTCAAG. Subsequently, it was cloned via the restriction sites *Nco*I and *Xho*I into the pCS2+MT vector. For bacterial expression of Glutothione-S-transferase- (GST-) tagged YopM, YopM was amplified from YopM in pACYC184 using the primers Forward AGGATCCATGTATGGTTTTGTTTGCAATGAA and Reverse GAAGCTTTCTACTCAAAAACATCATCTTCAAG and subsequently cloned into the pGEX-KG vector [40] via the restriction sites *Bam*HI and *Hind*III. YopM-CBP-SBP in pACYC184 was created by amplification of a fragment of YopM with the primers ATTGATCCATATGAATTTGCTCAT and TTCTAGAGTACGCGGCCGCCTCAAAAACATCATCTTCAAG, digestion with *Nde*I/*Xba*I and ligation into YopM in pACYC184 which had also been digested with *Nde*I and *Xba*I. This resulted in a YopM without a stop codon and a 3′-*Not*I-restriction site. The tandem-affinity-tag containing the Calmodulin-Binding-Peptide (CBP) [41] and the Streptavidin-Binding-Peptide (SBP) [42], [43] were derived from the commercially available pNTAP-vector from the Interplay™ Tandem Affinity Purification system (Stratagene) via the following cloning steps. Stop codons (oligo F: GATCCTAATTAATTAAG; oligo-R: GATCCTTAATTAATTAG) were inserted into the *Bam*HI site of pNTAP. Afterwards, an *Xba*I-linker (New England Biolabs) was inserted into the EcoRV site of pNTAP. The resulting pNTAP and the YopM-pACYC184 construct without stop codon and the additional *Not*I site described above were then digested by *Not*I and *Xba*I and the insert from pNTAP was ligated into the YopM-pACYC184 construct resulting in a YopM C-terminally tagged with the two consecutive affinity tags CBP and SBP from the pNTAP vector.

#### Chemicals

MEK1/2-inhibitor (SL327; Z-& E-a-(Amino-((4-aminophenyl)thio)methylene)-2-(trifluoromethyl)benzeneacetonitrile) and HA1077 were all purchased from Calbiochem, rotenone, 2-deoxyglucose and Flag-sepharose were purchased from Sigma.

### Cell culture, transfection, ATP-Depletion and infection

HEK293T (ATCC# CRL-11268) cells were grown on 100 mm or 35 mm tissue culture dishes (Nunc) at 37°C and 5% CO_2_ in DMEM with 10% FCS containing 100 IU/ml Penicillin and 100 µg/ml Streptomycin (all from Gibco). Transfection was carried out at approximately 50% confluence using the Calcium phosphate method following standard procedures. During serum starvation conditions HEK293T cells were incubated in DMEM as described above without FCS for 12 hours. For serum starvation medium was exchanged 16–24 hours before harvesting to serum-free DMEM with 100 IU/ml Penicillin and 100 µg/ml Streptomycin. For MEK1/2 inhibition 48 hours after transfection HEK293T cells were serum-starved and 16–24 hours later were treated with 6 µM MEK-inhibitor (see above under Chemicals). 60 minutes later cells were harvested. For ATP-depletion of HEK293T cells the medium was exchanged for PBS (Gibco) containing 10 mM 2-deoxyglucose and 5 µM rotenone (both from Sigma) [44]. J774A.1 (ATCC# TIB-67) cells were grown in RPMI1640 supplemented with 10% FCS, 100 IU/ml Penicillin and 100 µg/ml Streptomycin and additional 2 mmol Glutamine (all from Gibco). Upon infection medium of J774A.1 cells were changed to antibiotic free RPMI1640.

*Yersinia strains and Infection* – *Yersini*a *enterocolitica* strains used in this study are deltaYopM, a derivative of the *Yersinia enterocolitica* Serotype O:8 strain WA-314 harbouring the virulence plasmid pYVO8 [45], in which the YopM gene had been replaced by a kanamycin resistance cassette [10], the same deltaYopM strain complemented with the DNA construct YopM-CBP-SBP in pACYC184 (described under *plasmids*) (deltaYopM(pYopM-CBP-SBP)), deltaYopM complemented with YopM in pACYC184 [39] (deltaYopM(pYopM)), WA-C(pTTSS), the virulence plasmid cured *Yersinia enterocolitica* strain WA-C [45] harbouring the plasmid pTTSS encoding the TTSS secretion/translocation apparatus of WA-314 but no Yop effector gene [39] and WA-C(pTTSS) complemented with YopM in pACYC184 (WA-C(pTTSS+pYopM)). For infection, *Yersinia* cultures were grown overnight at 27°C and then diluted 1∶20 in fresh Luria-Bertani broth and grown for another 2 h at 37°C in order to induce activation of the *Yersinia* type III secretion machinery and expression of Yops. Bacteria were centrifuged and resuspended in ice-cold PBS. J774A.1 cells were then infected by addition of bacteria to the antibiotic free medium at a multiplicity-of-infection of about 50∶1. 90 min after infection of cells the bacteria were killed by the addition of gentamicin (100 µg/ml). Cells were harvested and lysed in phosphatase inhibition buffer (PBS, 25 mM beta-glyerophosphate, 1 mM sodiumfluoride, 1 mM Orthovanadate, 1x Complete protease inhibitor cocktail (Roche)) at the indicated time points.

### Tandem Affinity purification

Ten 100 mm culture dishes of J774A.1 cells (approximately 1×10^8^ cells) were infected with deltaYopM(pYopM-CBP-SBP) as described above. 90 min after infection the cells were washed 3 times with PBS and were then harvested and lysed in 10 ml of the lysis buffer supplied with the Interplay™ Tandem Affinity Purification kit (Stratagene) by three freeze-thaw rounds. The following tandem affinity purification procedure was performed according to the instructions of the manufacturer. Briefly, the lysate was cleared by centrifugation and then incubated with streptavidin sepharose for 2 hours at 4°C under constant rotation, washed three times with the provided streptavidin binding buffer and was then eluted with streptavidin elution buffer. This eluate was then incubated with calmodulin sepharose for another 2 hours at 4°C and subsequently washed again three times with calmodulin binding buffer. The washed beads were boiled for 10 min in 2x SDS-loading buffer and loaded onto a SDS-PAGE. Afterwards, the gel was Coomassie stained and the visible bands cut out and analyzed by mass spectrometry.

### Mass spectrometry

The proteins (bands cut out from Coomassie stained SDS gels) were reduced with 10 mM dithiothreitol (DTT) at 56°C for 30 min, the cysteine residues modified with iodacetamid (55 mM, ambient temperature, 20 min in the dark) and the protein in-gel digested with trypsin (conditions: 5 ng trypsin/µl (sequencing grade modified trypsin, Promega, Madison, USA) in 50 mM NH_4_HCO_3_, 37°C, 16 h). After digestion the gel pieces were repeatedly extracted (50% acetonitrile/5% formic acid), the combined extracts dried down in a vacuum concentrator and redissolved in 5% methanol/5% formic acid. 0.5 µl of sample was mixed with an equal volume of matrix solution (saturated solution of cyano-4-hydroxycinnamic acid (HCCA) in 65% water/35% acetonitrile/0.1% trifluor acetic acid (TFA)) and applied onto a MALDI target by the dried-droplet method. Peptide mass fingerprint data were determined on a MALDI-TOF mass spectrometer (REFLEX IV, Bruker Daltonics, Bremen, Germany) in reflector mode. Database searches were done with the Mascot search algorithm version 2.2 (Matrix Sciences, London, UK) using the following parameter: mass tolerance: 50 ppm, one missed tryptic cleavage allowed, fixed modification: carbamidomethyl cysteine, variable modification: monooxidized methionine, database searched: NCBI nr 20100116, searches limited to *mus musculus*.

### Bacterial protein expression, purification and pull-down assay

Bacterial protein expression and purification were done essentially as described [46]. Briefly, the empty pGEX-KG or YopM in pGEX-KG were transformed into BL21 E. coli. 20 ml overnight cultures were diluted in 500 ml LB medium and grown under constant shaking at 37°C to an OD of about 0.6. Then isopropyl thio-β-d-galctoside (IPTG) was added to a final concentration of 1 mM and cultures were incubated for further four hours. Bacteria were then harvested by centrifugation, resuspended in PBS with 1x Complete protease inhibitor cocktail (Roche) and lysed by ultrasonification. After pelleting the bacterial debris by centrifugation, the supernatant was aliquoted and stored at −80°C. Bacterial lysates were then used either for purification or for pull-down assays. In order to purify GST or GST-YopM bacterial lysates were poured onto a column loaded with 0.5 ml Glutathion-sepharose resin (GE-Healthcare). After binding the column was washed with 10 ml PBS +1% Triton-X100 and subsequently with 10 ml PBS. Proteins were eluted from the sepharose in 5 ml 100 mM NaCl, 50 mM Tris pH 7.5, 20 mM Glutathion (Sigma) and 1x Complete protease inhibitor cocktail (Roche). Protein amounts in the eluates were quantified by DC protein assay (Biorad). GST-pull-down assays were essentially done as described [47]. 50 µL of a 50% slurry Glutathion-sepharose was loaded with either GST or GST-YopM. After washing of the loaded sepharose and resuspension in binding buffer (PBS, 1% NP40, 1x Complete protease inhibitor (Roche)), 5 µl of [^35^S]-methionine (Hartmann Analytic GmbH, Braunschweig, Germany) labeled in vitro translated (TNT coupled transcription/translation system from Promega GmbH, Mannheim) proteins were added. After overnight incubation, the sepharose was washed eight times in binding buffer, boiled in 2x SDS-loading buffer and loaded onto a SDS-PAGE. Bound proteins were visualized by autoradiography.

### Western blot analysis

Cellular lysates were prepared by three consecutive freeze/thaw cycles in phosphatase inhibition buffer (PBS, 25 mM beta-glyerophosphate, 1 mM sodiumflouride, 1 mM Orthovanadate, 1x Complete protease inhibitor cocktail (Roche)). Lysates were then resolved in 10% SDS polyacrylamide gels and afterwards, proteins were transferred to PVDF membranes by electroblotting. Membranes were blocked in 5% milk powder in PBS +0.1% Tween for 1 h and subsequently incubated with the indicated first antibodies diluted in blocking solution overnight at 4°C. After washing three times in PBS +0.1% Tween, membranes were incubated in the appropriate second antibody for 2 h at room temperature, washed three times as above and bands were visualized by the ECL system (GE Healthcare). For the quantification of Westernblots femtoLUCENT™ PLUS-HRP (G-Biosciences) was used as chemiluminescence reagent. Chemiluminescence was recorded with the LAS 4000 mini Luminescent Image Analyzer (Fujifilm Corporation) and signals were analyzed using the Multi Gauge V3.2 software.

### Immunoprecipitation

HEK293T cells were co-transfected with the indicated plasmids. Cells were lysed 48 hours after transfection in PBS +1% Triton X-100 supplemented with Complete protease inhibitor cocktail by three consecutive freeze/thaw cycles. After clearing of the lysate by centrifugation lysates were incubated with 20 µl Flag-sepharose (Sigma) overnight. Afterwards the sepharose was washed eight times with 1 ml lysis buffer and then resuspended in 2x SDS-buffer, boiled and loaded onto SDS-PAGE. After western blotting, the membrane was incubated with the indicated antibodies.

### In vitro dephosphorylation assay

50 ng active RSK, ERK2 and MEK1 (Millipore) were preincubated with 1 µg of bacterially expressed GST or GST-YopM in lambda phosphatase buffer (New England Biolabs) supplemented with Complete protease inhibitor cocktail (Roche) in a total volume of 20 µl for 15 minutes on ice. Subsequently, 2000 units (0.5 µl) lambda phosphatase (New England Biolabs) were added and the reactions were incubated for 5 min for p-S380RSK and 60 min for p-S221RSK, ERK2 and MEK1 at 30°C under constant shaking. Reactions were stopped by adding 20 µl 2x SDS buffer and boiling for 10 min. 20 µl of each reaction were then loaded onto a SDS-PAGE and analyzed by western blotting.

## Results

### YopM interacts with all PKN and all RSK isoforms

**Figure 1:**
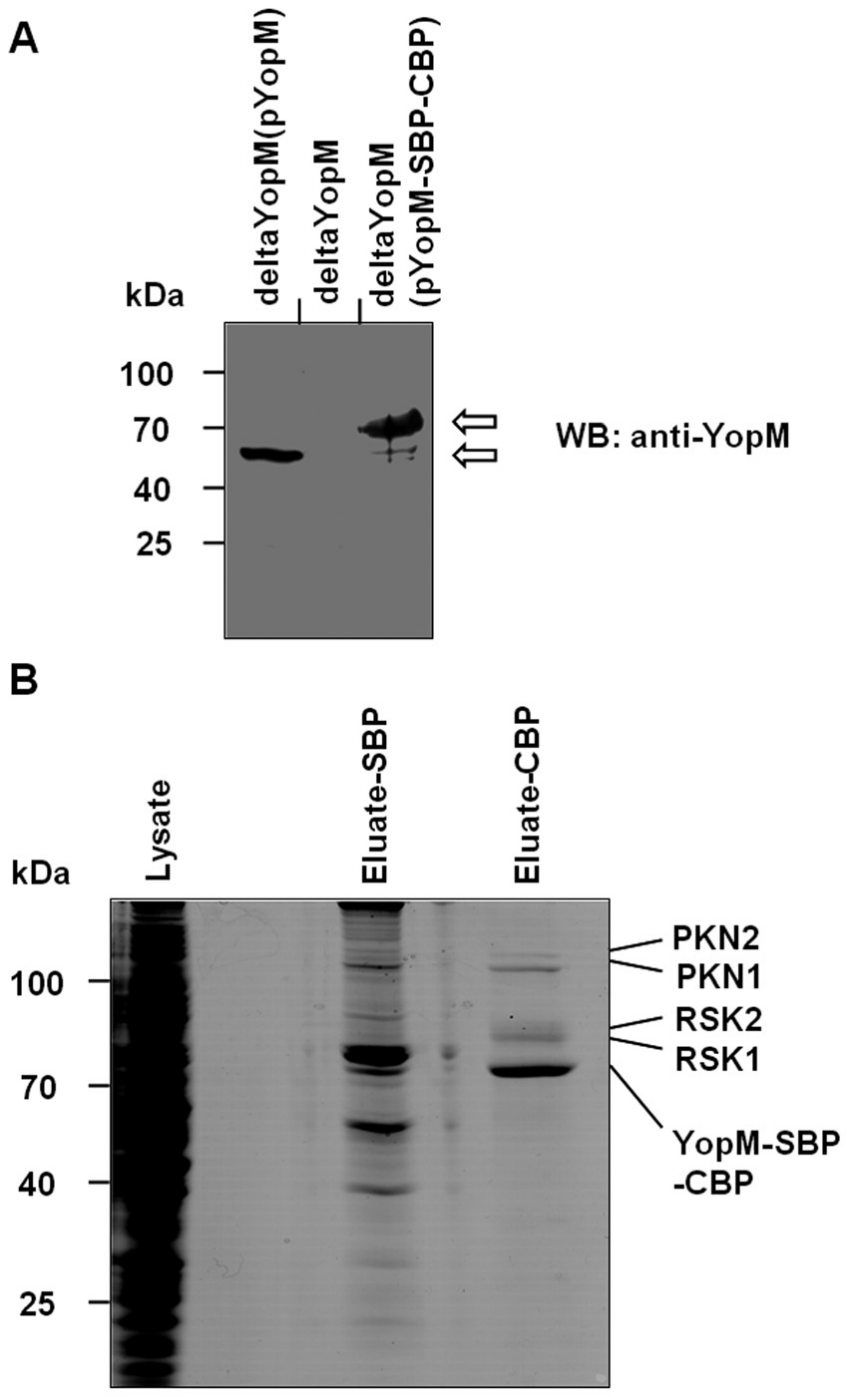
Identification of YopM interacting proteins by tandem-affinity-purification. (A) HEK293T cells were infected with the indicated strains, lysed and extracts were subjected to western blotting with anti-YopM antibody. (B) J774A.1 cells were infected with deltaYopM(pYopM-CBP-SBP) and lysates were prepared 90 minutes after infection. After tandem affinity purification the beads were boiled and subjected to SDS-PAGE. Visible bands after Coomassie-staining were excised and subsequently analyzed by mass spectrometry.

A previous study identified PKN2 and RSK1 as interaction partners of YopM [23]. In order to verify these interaction partners under lifelike infection conditions and to potentially discover novel ones, we translocated affinity tagged YopM into the macrophage cell line J774A.1 via the TTSS of *Yersinia*. For this purpose the coding region of the *Yersinia enterocolitica* Serotype O:8 strain WA-314 YopM gene was fused N-terminally to a Tandem-affinity-Purification tag (TAP-tag), which consists of two separate affinity tags, one calmodulin binding peptide (CBP) and one streptavidin binding peptide (SBP), which can be specifically eluted from their affinity matrices with EGTA and Biotin, respectively. The fusion-construct was transformed into a *Yersinia enterocolitica* O8 strain in which the YopM gene had been deleted, WA-deltaYopM, resulting in the complemented strain WA-deltaYopM(pYopM-CBP-SBP) (Fig. 1A). This complemented strain was then used to infect J774A.1-cells. Lysates of the infected cells were first bound to a Streptavidin-sepharose-matrix and subsequently eluted. This eluate was then adsorbed to a Calmodulin-sepharose and eluted again. The eluate was separated by SDS-PAGE and Coomassie stained. The visible bands were cut out and analyzed by mass spectrometry (Fig. 1B). While no proteins were precipitated when cells were infected with the uncomplemented deltaYopM strain (not shown), several proteins were bound by YopM-CBP-SBP. Two members of the RSK family, RSK1 and RSK2, and two members of the PKN family, PKN1 and PKN2, were identified. Thus, besides confirming the already described interaction of YopM with RSK1 and PKN2, our screen recovered RSK2 and PKN1 as novel binding partners, which are closely related isoforms of RSK1 and PKN2. All proteins were identified by peptide mass fingerprint analysis resulting in very low expect values (Table 1). The interactions were also verified vice-versa. Following infection YopM-SBP-CBP not only pulled down RSK1 and PKN1 but was also efficiently co-immunoprecipitated with RSK1 and PKN1 (Fig. S1).

**Table 1:** Peptides identified by peptide mass fingerprint analysis.

Protein	Expect	Identified peptides
PKN1	2.9e-9	LKEGAENLR; EGAENLRR; SLAPVELLLR; KLLLTAQQMLQDSK; LLLTAQQMLQDSK; KAVSEAQEK; LGELPADHPK; NLPETIPWSPPPSVGA; SSLRGEAENATEVSTV; ELELAVFWR; LEDFLDNER; QMNIDVATWVR; SPLTLEDFK; SSGELFAIK; LDNLLLDTEGYVK; FLSAEAIGIMR; SLGWDVLLAR; RLPPPFVPTLSGR; LPPPFVPTLSGR; DARPLTAAEQAAFR
PKN2	9.1e-10	SSVVIEELSLVASPTLSPR; FTLELDR; ELEISVYWR; LEDFLDNQR; APQMNINIATWGR; ATSVALPGWSPSDNR; AIPTVNHSGTFSPQTP; LDFDLEPEPPPAPPP; VLDIPGQGSETVFNIE; SKSEYELSIPDSGRSCWGVGELD; SCWGVGELD; FQFSLQDFR; IFETVNSVR; FLSTEAISIMR; VKPPFVPTIR
RSK1	1.1e-13	EISITHHVK; VLGQGSFGK; KVTRPDSGHLYAMK; DILADVNHPFVVK; LHYAFQTEGK; DLKPENILLDEEGHIK; LTDFGLSK; LGMPQFLSTEAQSLLR; LGSGPDGAEEIKR; HIFYSTIDWNK; DSPGIPPSAGAHQLFR; GFSFVATGLMEDDGKP; TTQAPLHSVVQQLHGK; RDPSEEIEILLR; YGQHPNIITLK; HVYLVTELMR; QKFFSER; TVEYLHSQGVVHR; DLKPSNILYVDESGNPECLRICDFGFAK; FTLSGGNWNTVSETAK; MLHVDPHQR; QVLQHPWITQK; DKLPQSQLSHQDLQLVK
RSK2	1.4e-6	VVGVGVGVELALGGAAAAR; DILVEVNHPFIVK; DLKPENILLDEEGHIK; LGMPQFLSPEAQSLLR; LGAGPDGVEEIKRHSFFSTIDWNK; DSPGIPPSANAHQLFR; RDPTEEIEILLR; TVEYLHAQGVVHR; NQSPVLEPVGR

**Figure 2:**
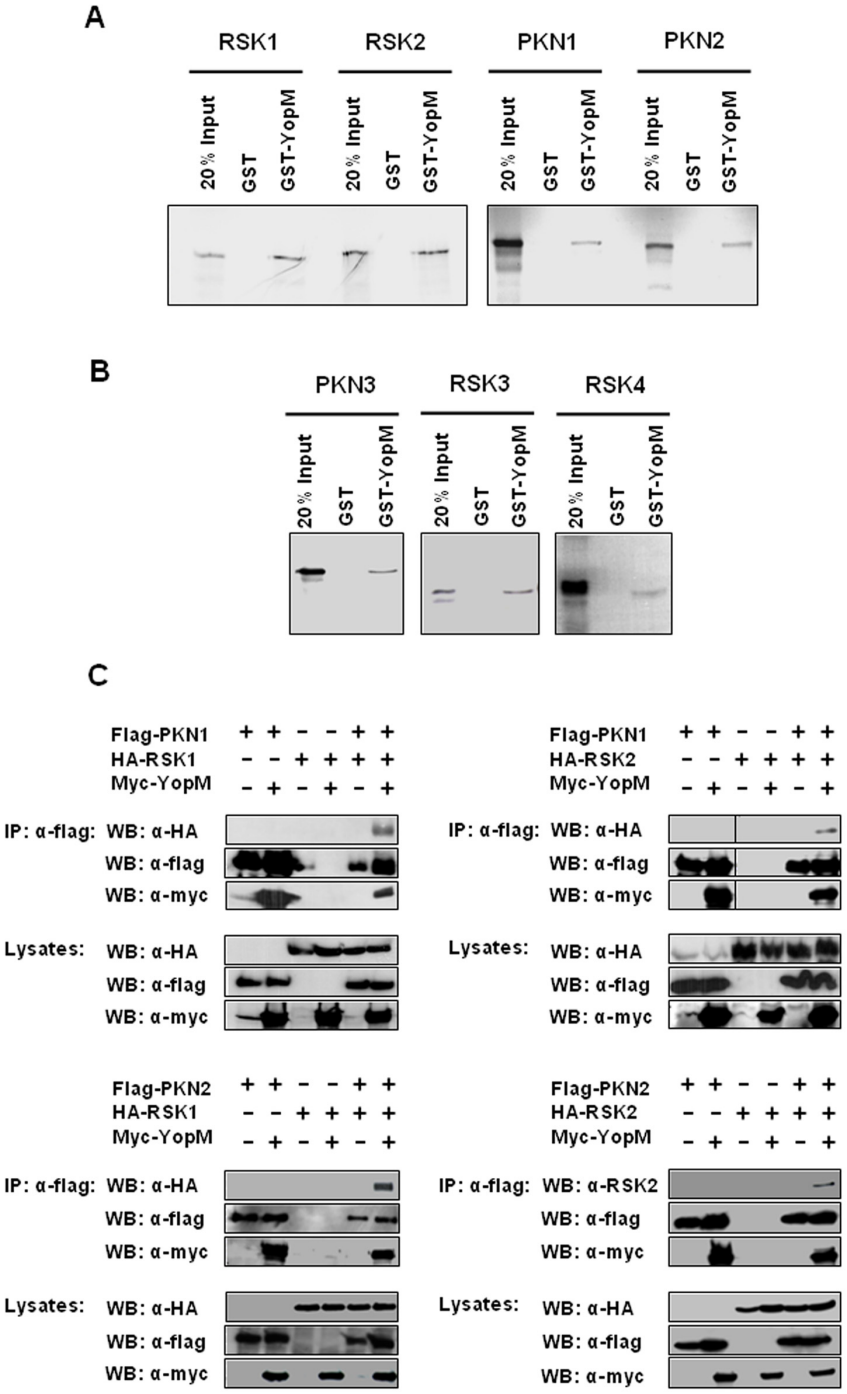
Analysis of the YopM complex. (A) Interactions were confirmed individually by GST-pull-down assays with [^35^S]-methionine labeled in vitro translated proteins. (B) Interactions between YopM and further RSK and PKN isoforms not recovered by Tandem affinity purification, RSK3, RSK4 and PKN3, were analyzed by pull-down assays as in (A). (C) HEK293T cells were transfected with the indicated plasmids and harvested after 48 hours. Lysates were then incubated with 20 µl of Flag-sepharose and the precipitates and aliquots of the lysates before immunoprecipitation were analyzed by western blotting with the indicated antibodies.

In order to further confirm the interaction of the identified proteins with YopM GST-pull-down assays were performed. Bacterially expressed GST or GST-YopM immobilized to Glutathione-sepharose were separately incubated with in vitro translated [^35^S]-methionine labeled PKN1, PKN2, RSK1 or RSK2. All labeled proteins bound to GST-YopM but not to GST alone (Fig. 2A) indicating a direct interaction with YopM. Our finding that YopM interacts with two isoforms of each kinase family prompted us to investigate whether YopM also binds to the other members of the RSK and PKN families. Indeed, in GST pull-down assays we found binding of YopM to PKN3, RSK3 and to RSK4 (Fig. 2B). These findings indicate that YopM interacts with all known members of the RSK and PKN family of kinases.

It was shown that YopM assembles PKN2 and RSK1 into one complex [23]. As we discovered that YopM interacts all with known members of the RSK- and PKN families we sought to analyze whether these proteins are assembled in arbitrary combinations or whether preferred combinations exists. Thus, we analyzed interactions between the kinases shown to associate with YopM in J774A.1 cells according to our tandem affinity purification assay. First, we asked whether PKN1 can co-immunoprecipitate both RSK1 and RSK2 in the presence of YopM and whether the same is true for PKN2. As shown in Figure 2C YopM can be robustly co-immunoprecipitated with PKN1 and PKN2. Furthermore, RSK1 (left upper panel) and RSK2 (right upper panel) could separately be co-immunoprecipitated with PKN1 in the presence but not in the absence of YopM. The same results were obtained when co-immunoprecipitation with PKN2 was assayed (left lower and right lower panel). These experiments confirm the earlier finding that PKN isoforms physiologically do not interact with RSK. Interaction occurs only if YopM is present in the cell. Furthermore, there does not seem to be a preference of one PKN isoform for a specific RSK isoform. These results suggest that YopM forms complexes of RSK and PKN subunits in an arbitrary combination.

### YopM inhibits deactivation of RSK in the absence of ERK signalling

**Figure 3:**
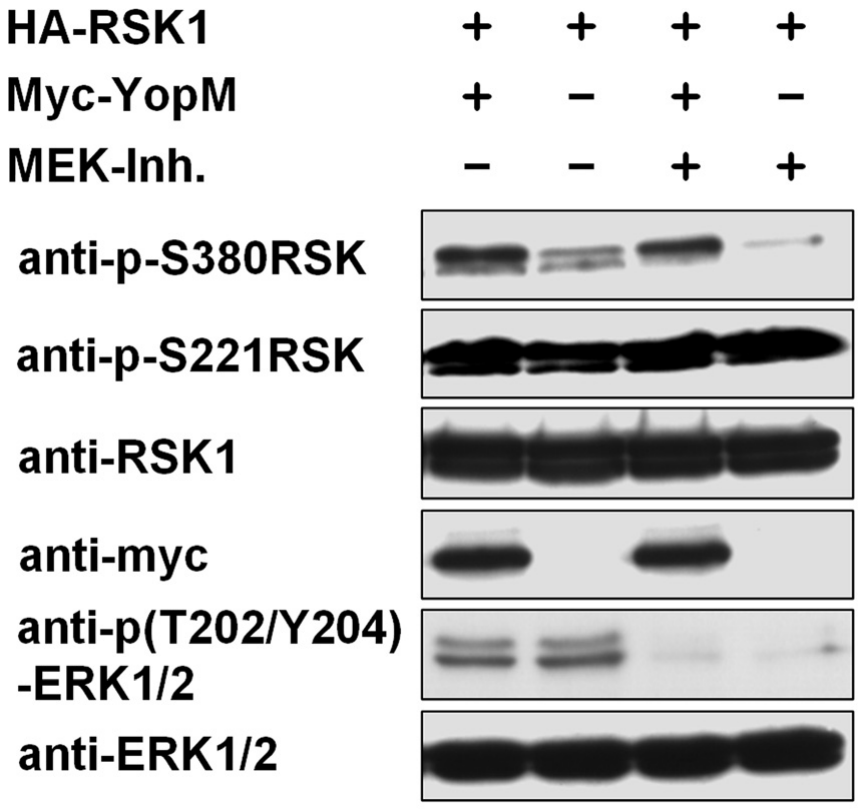
YopM induces RSK activation in the absence of ERK signalling. HEK293T were transfected with the indicated plasmids. 48 hours after transfection cells were serum-starved and 16–24 hours later were treated with 6 µM MEK-inhibitor. 60 minutes later cells were lysed and the lysates subjected to western blotting with the indicated antibodies.

YopM has been described to induce activation of RSK in kinase assays, yet the underlying mechanism has not been further investigated [23]. We determined in YopM- versus empty vector-transfected serum-starved HEK293T cells the activation status of RSK by analyzing phosphorylation at serine 380, which correlates well with kinase activity [48], and phosphorylation at serine 221 in the activation loop which is also critically important for kinase activity [49], [50]. We found significant activation of the MEK-ERK-RSK pathway in our HEK293T cells as exemplified by strong basal phosphorylation of ERK1/2 at threonine 202/tyrosine 204 and RSK at serine 380 and serine 221 also under serum starvation conditions (Figure 3). Though the phosphorylation status of ERK did not differ between YopM and empty vector transfected cells, RSK was hyperphosphorylated at serine 380 when YopM was present. When MEK inhibitor was added to the medium ERK1/2 and RSK serine 380 were rapidly dephosphorylated in empty vector transfected cells. Quantification revealed that phosphorylation of serine 380 of RSK was reduced by 60+/−14% in one hour in these cells (mean value and standard deviation was calculated from 8 measurements from three independent experiments). In contrast, RSK phosphorylation at serine 380 in the YopM transfected cells declined much slower, only 23+/−13% in one hour, although ERK was as rapidly dephosphorylated as in the empty vector transfected cells. Thus, RSK remains phosphorylated at serine 380 in the presence of YopM even when activating upstream signals from MEK-ERK are inhibited. Phosphorylation at serine 221 was unaltered by the presence of YopM or the addition of MEK inhibitor. This may indicate a slower phosphorylation/dephosphorylation turnover at this site. Thus, YopM induces hyperactivation of RSK without altering ERK activity and inhibits deactivation of RSK in the absence of upstream ERK signalling.

### YopM inhibits dephosphorylation of RSK

**Figure 4:**
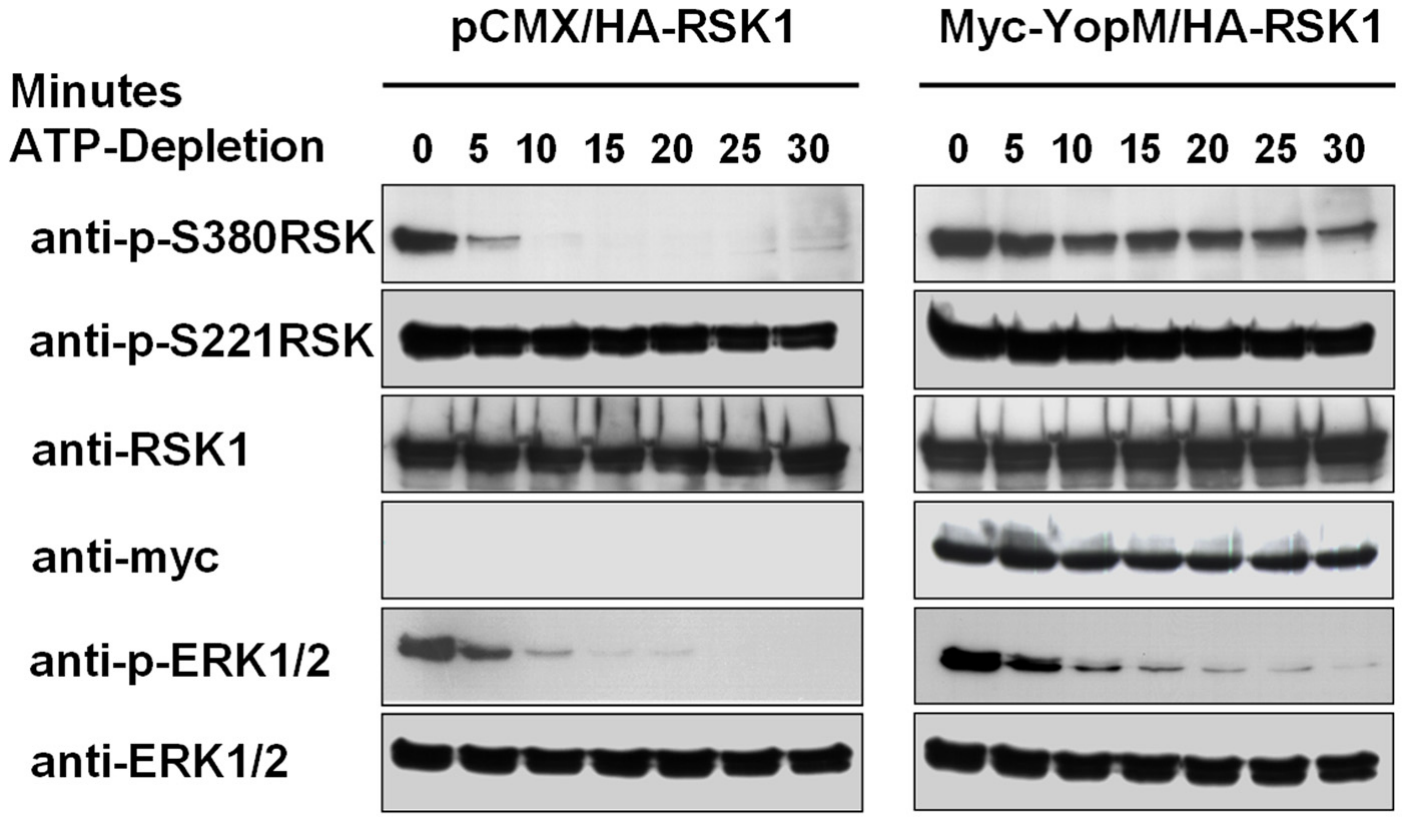
YopM protects RSK from dephosphorylation during ATP-depletion. 48 hours after transfection of HEK293T with the indicated plasmids, the medium of the cells was exchanged with PBS supplemented with rotenone and 2-deoxyglucose. Cells were then harvested in 5 minute intervals and flash frozen. Westernblots of the lysates were then analyzed with the indicated antibodies.

In the absence of YopM ERK1/2 and RSK obviously become rapidly and thoroughly dephosphorylated and thereby inactivated when upstream MEK signalling was inhibited indicating tight regulation of the MEK-ERK-RSK pathway by phosphatases. We hypothesized that the sustained phosphorylation of RSK at serine 380 caused by YopM might result from impaired dephosphorylation, maybe through protection from phosphatases. To further investigate this idea, we performed ATP-depletion assays. Due to inhibition of glycolysis by 2-deoxyglucose and the respiratory chain by rotenone as well as by exchanging the medium for glucose free PBS intracellular ATP is consumed by the cell but not replenished [44]. In vector transfected HEK293T cells, we observed a rapid dephosphorylation of ERK1/2 and RSK at serine 380 after induction of ATP-depletion conditions indicating again a rather fast turnover at these phosphorylation sites (Figure 4, left panel). Serine 380 of RSK was completely dephosphorylated after 5–10 minutes. In contrast, phosphorylation of serine 380 in YopM transfected cells remained stable for at least 30 minutes with only minor dephosphorylation (Figure 4, right panel). In parallel ERK1/2 was dephosphorylated comparably as in vector transfected cells, indicating that the YopM effect on dephosphorylation inhibition is specific for RSK and independent from upstream signalling. As seen before, phosphorylation declined very slowly if at all at serine 221 under these conditions, consistent with a slow dephosphorylation kinetic. Addition of HA1077, an inhibitor of PKN, prior to ATP-depletion did not reverse the effects of YopM (data not shown). These results strongly suggest that YopM protects phosphorylated RSK from the dephosphorylation by phosphateses.

**Figure 5:**
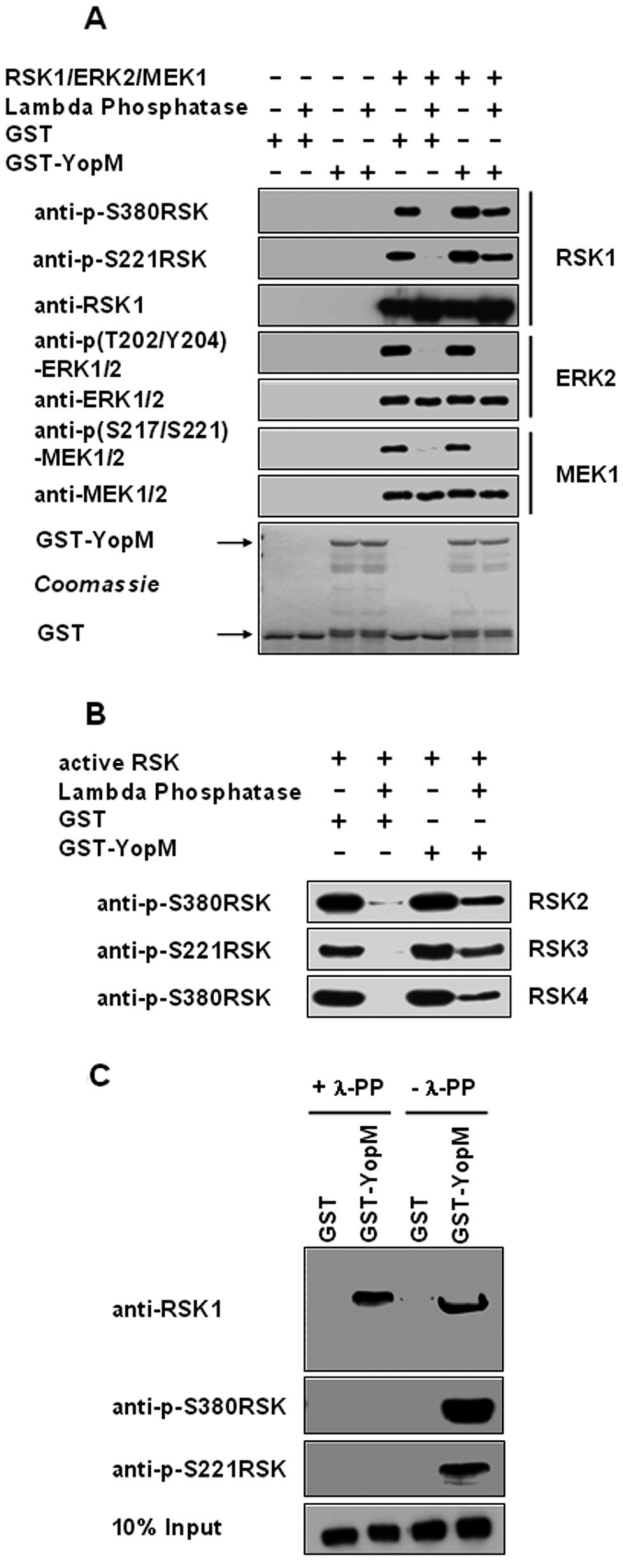
YopM specifically protects RSK from dephosphorylation during in vitro dephosphorylation assays. (A) 50 ng active RSK1 and 1 µg GST or 1 µg GST-YopM were preincubated in lambda-phosphatase buffer for 15 minutes on ice. Then 1 µl of lambda phosphatase was added and the reaction was transferred to 30°C under gentle constant shaking. The reaction was stopped by addition of 2x SDS buffer and boiling. After western blotting analysis was carried out with the antibodies indicated. (B) As in (A) except that RSK2, RSK3 and RSK4 were used in the reaction. (C) YopM interacts with both phosphorylated and unphosphorylated RSK1. 500 ng active RSK was pretreated for 90 minutes with lambda phosphatase, while 500 ng was left untreated. 250 ng of the dephosphorylated or the active RSK1 was then incubated with GST or GST-YopM bound to Glutathione-sepharose and processed as described under pull-down in the Experimental Procedures section. After washing the beads were boiled in 2x SDS buffer and subjected to western blotting with the antibodies stated.

In order to further investigate whether YopM directly protects RSK from dephosphorylation we performed in vitro dephosphorylation assays with purified defined components. Therefore we incubated purified commercially available active RSK1 (pre-phosphorylated by active ERK1/2 and PDK1) with lambda phosphatase in the presence of bacterially expressed and purified GST or GST-YopM. RSK1 was readily dephosphorylated by lambda phosphatase on serine 380 and serine 221 when GST was added to the reaction mix (Fig. 5A). In sharp contrast, when GST-YopM was added dephosphorylation of serine 380 and serine 221 by lambda phosphatase was effectively inhibited. This effect of YopM was specific for RSK as neither ERK2 nor MEK1 were protected by YopM from dephosphorylation by lambda phosphatase. We also tested whether RSK2, RSK3 and RSK4 are protected from dephosphorylation by YopM by analysing phosphorylation at serine 380 in RSK2 and RSK4 and at serine 221 in RSK3. All RSK isoforms were efficiently dephosphorylated by lambda-phosphatase in the presence of GST while GST-YopM clearly inhibited this process (Fig. 5B). These findings further corroborate the conclusions from the ATP-depletion and the MEK inhibition experiments, that YopM inhibits dephosphorylation of activated RSK isoforms. These results also prompted us to ask whether YopM might specifically interact with phosphorylated RSK. We performed GST-pull-down assays with active RSK1 (see above) which was either left untreated or was pretreated with lambda phosphatase for 90 minutes, the latter resulting in complete dephosphorylation. We could clearly show that YopM interacts with both phosphorylated and unphosphorylated RSK1 (Fig. 5C). Thus interaction with YopM is independent from the phosphorylation status of RSK.

### Translocation of YopM during infection inhibits RSK dephosphorylation after initial activation

Lastly, we analyzed whether we could also find an influence of YopM on the RSK phosphorylation status in J774A.1 cells during infection. We used four different *Yersinia* strains for these experiments. Two strains express modules constituting a functional type-three-secretion system, without (WA-C(pTTSS)) or with YopM complementation (WA-C(pTTSS+pYopM)). The other two strains were the YopM-deleted strain WA-deltaYopM and WA-deltaYopM complemented with YopM (WA-deltaYopM(pYopM)). Using backgrounds which either express other Yops (WA-deltaYopM) or not express any other Yops (WA-C(pTTSS) served to investigate whether other Yops influence the effects of YopM. Furthermore, the two different backgrounds WA-C(pTTSS) and WA-deltaYopM were used to overexpress YopM as both WA-C(pTTSS+pYopM) and WA-deltaYopM(pYopM) express YopM from a plasmid. This resulted in an about five-fold overexpression of YopM compared to wildtype strain WA-P as determined by westernblot quantification of lysates from infected cells (also demonstrated in Figure S2). This enabled us to observe YopM mediated effects on RSK phosphorylation more clearly than with a wildtype strain.

**Figure 6:**
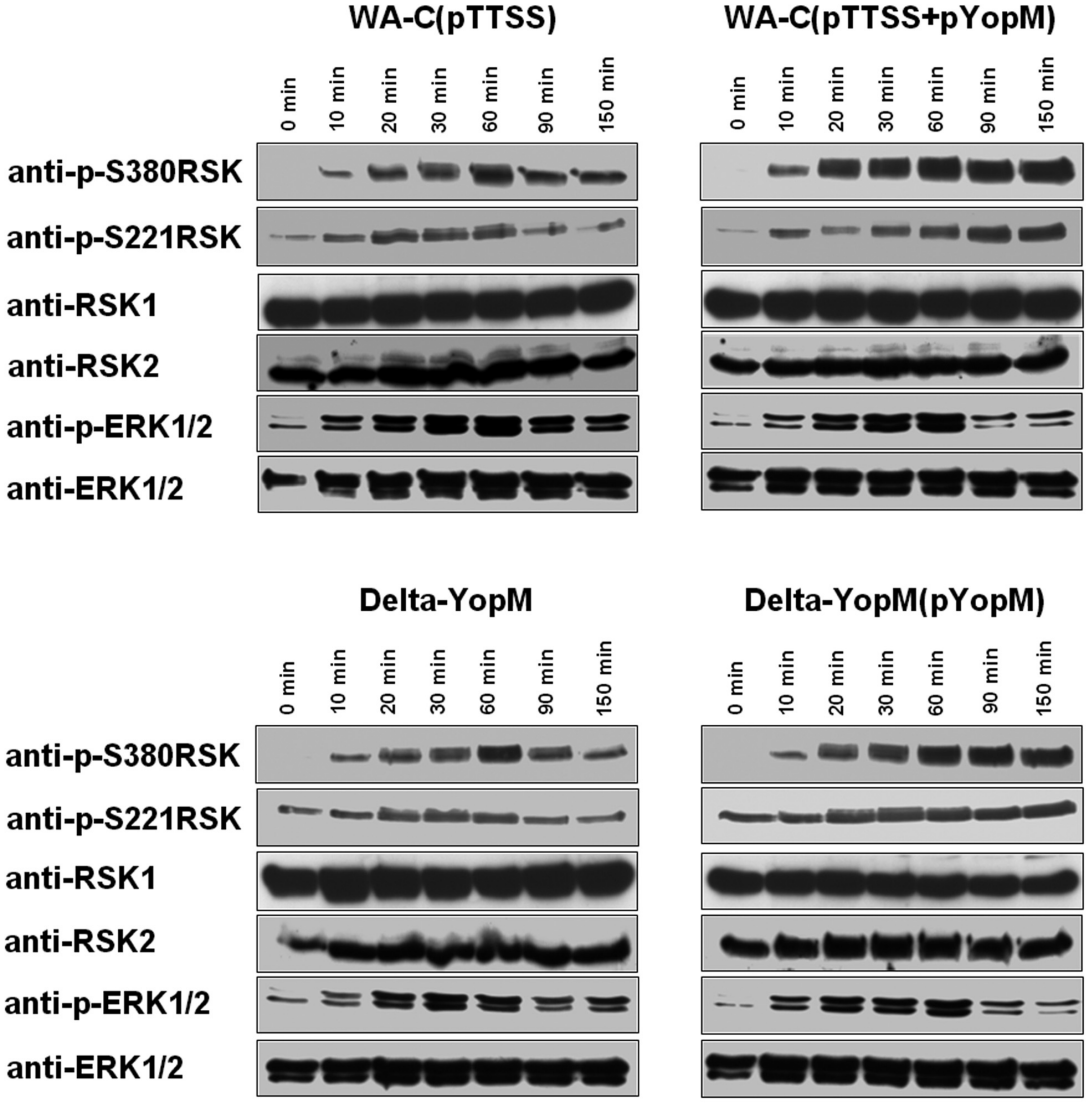
YopM delivered during infection induces protracted activation of endogenous RSK in J774A.1 cells. J774A.1 cell were infected with WA-C(pTTSS) (left upper panel) or with WA-C(pTTSS+pYopM) (right upper panel) or with WA-deltaYopM (left lower panel) or with WA-deltaYopM(pYopM) (right lower panel) and harvested at the indicated time points. Bacteria were killed by the addition of gentamicin after 90 minutes. Cellular lysates were then subjected to western blotting and probed with the indicated antibodies.

In contrast to the HEK293T cells, basal serine 221 and serine 380 phosphorylation levels of RSK in J774A.1 cells were low under resting conditions (Fig. 6). Upon infection with each of the four *Yersinia* strains RSK phosphorylation at serines 380 and 221 rapidly increased with a maximum at about 30–60 minutes after infection. In cells infected with the strains WA-C(pTTSS) (Fig. 6, left upper panel) and WA-deltaYopM (Fig. 6, left lower panel) serine 380 and serine 221 phosphorylation declined progressively after 60 minutes. The decrease of phosphorylation of RSK was parallel to the decrease of ERK-phosphorylation without detectable delay confirming tight coupling of ERK and RSK activation. In contrast, in J774A.1 cells infected with *Yersinia* strain WA-C(pTTSS+pYopM) (Fig. 6, right upper panel) and WA-deltaYopM(pYopM) (Fig. 6, right lower panel) phosphorylation of RSK remained at a constant level indicating impaired dephosphorylation and deactivation of RSK. At the same time decrease of ERK phosphorylation proceeded with the same kinetics as in cells infected with YopM-negative *Yersinia* strains indicating a YopM-dependent uncoupling of RSK activation/phosphorylation from ERK1/2 activation. These results show that also under infection conditions YopM induces protracted RSK activation/phosphorylation independent of ERK signalling. In general we did not observe major differences between cells infected with WA-deltaYopM or WA-C(pTTSS) backgrounds indicating that other Yops do not influence YopM effects on RSK phosphorylation. Noteworthy, under our experimental condition we did not observe significant inhibition of ERK1/2 by YopP, which is present in the WA-deltaYopM but not in the WA-C(pTTSS) background. Competition for translocation due to overexpression of YopM may be one explanation, though we still observed YopP-induced apoptosis at later time points in the J774A.1 cells indicating YopP translocation at effective levels. Also, different levels of phagocytosis resistance obviously do not result in differences between the WA-deltaYopM or WA-C(pTTSS) backgrounds, probably due to the rapidity of type three secretion mediated effector injection.

Taken together, we conclude from the experiments described above that YopM protects RSK from dephosphorylation leading to its sustained activation.

## Discussion

All intracellular Yops exert their effects on the cell by interacting with cellular proteins and modifying their activity [5]. The aim of this study was to identify interacting eukaryotic proteins of YopM under conditions which match the physiological situation during infection as closely as possible. Therefore, we tagged YopM C-terminally with two affinity tags to enable two consecutive affinity purification steps. This allows purification of larger complexes as no harsh elution conditions have to be employed and results in low background contamination [51]. Furthermore, our approach allows purification of proteins which bind to YopM during infection, as in our experiments YopM is translocated via the TTSS into the cell. By this, we identified four kinases, RSK1 and 2 and PKN1 and 2, as binding partners of YopM. Two of the proteins, RSK1 and PKN2, have previously been described as interacting partners of YopM [23]. Thus, our study identifies additional YopM-interacting proteins and confirms previous findings. Therefore, RSK and PKN family members are likely to be major host cell mediators of YopM action. This is in line with two very recent reports, which find reduced virulence of *Yersinia* strains deficient for binding to RSK1 [52], [53]. Several factors may contribute that we recovered more interaction partners than previous studies [23], [52], [53]. Beside methodological differences (use of different cell lines, mode of YopM delivery, method of purification) there is considerable variability between different YopM proteins from different species and serogroups. While previous investigations used YopM proteins with rather low LRR numbers of 13–15 units, our YopM harbours 20 LRRs. Furthermore, YopM proteins differ in their composition of different LRRs, which are mostly 20 amino acids but some also 21 and 22 amino acids long with our YopM having only one 22 amino acid LRR (LRR 4). This may give rise to divergent binding sites in particular for PKN members, as PKN2 has been found to interact with an internal region of YopM comprising LRR 6–15 [53]. Moreover, although the C-terminal 6 amino acids of YopM required for RSK1 binding are highly conserved other regions of the YopM protein might contribute to the RSK binding specificity. Thus, it currently remains to be determined whether all YopM proteins are able to bind several members of the RSK and PKN families.

The finding that YopM interacts with all members of the RSK and the PKN family raises the question whether YopM exploits the physiological functions of these kinases or whether these kinases are hijacked and directed towards novel functional contexts. With the exception of Rsk2, knockout mice have not been published for RSK or PKN family members, thus no definite statement can be made concerning redundancy, but some form of specificity for these kinases seems very likely. Thus, interaction of YopM with several isoforms may suggest, that no specific function of RSK may be targeted. Furthermore, up to now we did not find alterations in the phosphorylation status of diverse known endogenous targets of RSK (data not shown). PKN targets have not yet been investigated by us. Previous work suggested that PKN2 is not directly activated by YopM but indirectly by the overactivated RSK1 [23]. Different mechanisms are conceivable. One may be that PKN is directly phosphorylated by RSK. Another possibility might be activation by some steric mechanism as we were not yet able to detect hyperphosphorylation of PKN in YopM transfected cells (data not shown). It will be important to dissect this process in more detail in future studies. Modulation of RSK activity by YopM may have different consequences: Sustained activation of RSK by YopM may result in sustained phosphorylation of a specific subset of endogenous RSK targets, which we and others could not identify so far. Alternatively, activation of RSK may serve the only purpose of activating the PKN subunits of the YopM complex which subsequently phosphorylate physiological substrates. Lastly, activation of RSK and/or PKN may result in the phosphorylation of unphysiological substrates.

Interaction of YopM with RSK1 has been shown to activate RSK1 as analyzed by kinase assays [23]. This study uncovers the underlying molecular events resulting in sustained RSK activation. We find that YopM protects all RSK isoforms from being dephosphorylated *in vitro* and *in vivo*. It is currently not exactly known, which phosphatases are involved in the dephosphorylation of RSK, but the rapidity of dephosphorylation when upstream ERK signalling is interrupted suggests a tightly regulated system. Only two phosphatases, PP2Cdelta [54] and PP2A [55] have been described to interact with RSK. We were not able to block dephosphorylation of RSK under ATP-depletion conditions by addition of 50 nM okadaic acid (data not shown), which is an effective inhibitor of both PP2A and PP1. In our opinion this argues against a major role in vivo for PP2A in dephosphorylation of RSK. Members of PP2C family of phosphatases are notoriously unresponsive to commercially available phosphatase inhibitors [56], which may explain the insensitivity of the dephosphorylation to okadaic acid. Whether several PP2C members are involved in the regulation of RSK will have to be determined. The exact dynamics of phosphorylation and dephosphorylation of RSK as well as the identification of the factors involved will be an important area of research in the future.

It is currently unclear how yersiniae may benefit from sustained activation of RSK during infection. As already discussed above, activation of RSK may only serve the subsequent activation of PKN as we and others were so far unsuccessful in identifying changes in the phosphorylation status of some of the endogenous substrates of RSK ([23] and data not shown). RSK activation by YopM may be confined to the microenvironment of the YopM complex. Yet, although RSK has not been found to play a role during bacterial infections, there are a number of studies reporting virally induced pathologically sustained RSK activation during viral infection and replication [57], [58], [59]. These studies found sustained RSK activity to be important for replication of a broad range of different unrelated viruses like HIV, KSHV and vaccinia virus. Most importantly, the viral factor ORF45 from KSHV was identified to induce sustained RSK activation by a mechanism very similar to that of YopM [60]. ORF45 directly binds to RSK and obviously physically shields ERK and RSK from dephosphorylation which results in constant RSK activation. In CMV infection impaired dephosphorylation of ERK and p38 has been implicated in efficient viral gene expression [61], [62]. Thus, activation of ERK-RSK may be a common and potentially important incident during infection. However, none of the studies identified the targets downstream of RSK which mediate the effects of sustained activation or define the cellular events which are induced. We can currently not unambiguously demonstrate that overactivation of RSK is the critical molecular event responsible for YopM-conferred pathogenicity. Yet we believe on basis of our and the recent results of others [52], [53] that this is highly likely. We did not observe any other YopM-mediated on the kinases like inhibition, degradation or subcellular mislocalization (data not shown).

To summarize, in accordance with previous findings we identified the RSK and PKN families of kinases as the intracellular mediators of YopM effects. Moreover, we can clarify the mechanism how interaction with YopM results in over-activation of RSK with YopM shielding RSK from the action of phosphatases resulting in sustained activating phosphorylation. Protection of interacting proteins from dephosphorylation is a novel function of a bacterial effector, which may apply also for other leucine-rich-repeat containing proteins from other bacterial species related to YopM like SspH1 from *Salmonella* [63] and IpaH from *Shigella* [64]. Future studies will also have to address the physiological functions of RSK and PKN in the immune defense of the host during infection.

## Supporting Information

Figure S1 J774A.1 cells were left uninfected (lane 1 left and right panel) or were infected with deltaYopM(pYopM-CBP-SBP) (lanes 2–4 left and right panel)and lysates were prepared 90 minutes after infection. Lysates were then incubated with 20 µl of Streptavidine-sepharose (lanes 1 and 2 left and right panel) or Protein A/G sepharose (lanes 3 and 4 left and right panel) and the precipitates and aliquots of the lysates before immunoprecipitation were analyzed by western blotting with the indicated antibodies.
(0.57 MB PDF)

Figure S2 Analysis of YopM quantities translocated into J774.1 cells by different Yersinia strains. J774.1 cell were infected with WA-P, WA-deltaYopM, WA-deltaYopM(pYopM), WA-C(pTTSS) and WA-C(pTTSS+YopM) and harvested after 90 min. Cellular lysates were then subjected to western blotting and probed with the indicated antibodies.
(0.35 MB PDF)
