# Supplementary material for: Yersinia Virulence Factor YopM Induces Sustained RSK Activation by Interfering with Dephosphorylation
Source: PLoS One. 2010 Oct 5;5(10):e13165. doi: 10.1371/journal.pone.0013165 (PMC2950144; doi:10.1371/journal.pone.0013165)
Supplement: Figure S1 — J774A.1 cells were left uninfected (lane 1 left and right panel) or were infected with deltaYopM(pYopM-CBP-SBP) (lanes 2–4 left and right panel)and lysates were prepared 90 minutes after infection. Lysates were then incubated with 20 µl of Streptavidine-sepharose (lanes 1 and 2 left and right panel) or Protein A/G sepharose (lanes 3 and 4 left and right panel) and the precipitates and aliquots of the lysates before immunoprecipitation were analyzed by western blotting with the indicated antibodies. (0.57 MB PDF) [file pone.0013165.s001.pdf]

Suppl. Fig. S1

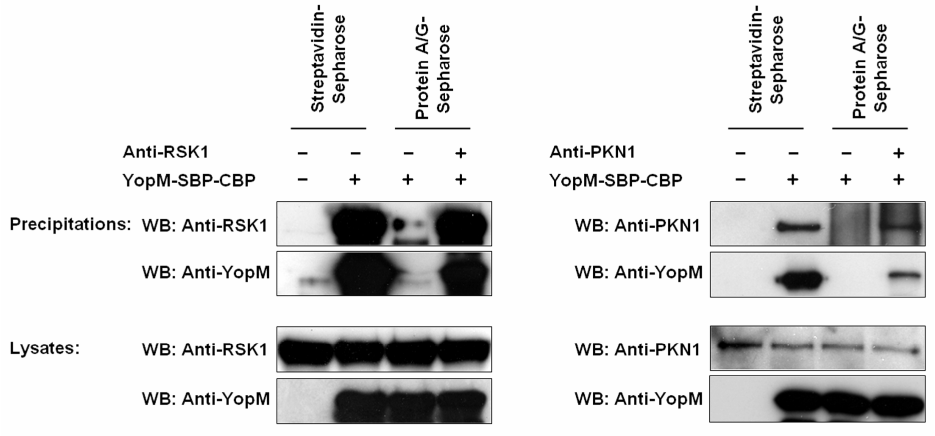

Supplementary Fig. S1: J774A.1 cells were left uninfected (lane 1 left and right panel) or were infected with deltaYopM(pYopM-CBP-SBP) and lysates were prepared 90 minutes after infection. Lysates were then incubated with 20 µl of Streptavidine-sepharose or Protein A/G sepharose and the precipitates and aliquots of the lysates before immunoprecipitation were analyzed by western blotting with the indicated antibodies.
