# Supplementary material for: Yersinia Virulence Factor YopM Induces Sustained RSK Activation by Interfering with Dephosphorylation
Source: PLoS One. 2010 Oct 5;5(10):e13165. doi: 10.1371/journal.pone.0013165 (PMC2950144; doi:10.1371/journal.pone.0013165)
Supplement: Figure S2 — Analysis of YopM quantities translocated into J774.1 cells by different Yersinia strains. J774.1 cell were infected with WA-P, WA-deltaYopM, WA-deltaYopM(pYopM), WA-C(pTTSS) and WA-C(pTTSS+YopM) and harvested after 90 min. Cellular lysates were then subjected to western blotting and probed with the indicated antibodies. (0.35 MB PDF) [file pone.0013165.s002.pdf]

Suppl. Fig. S2

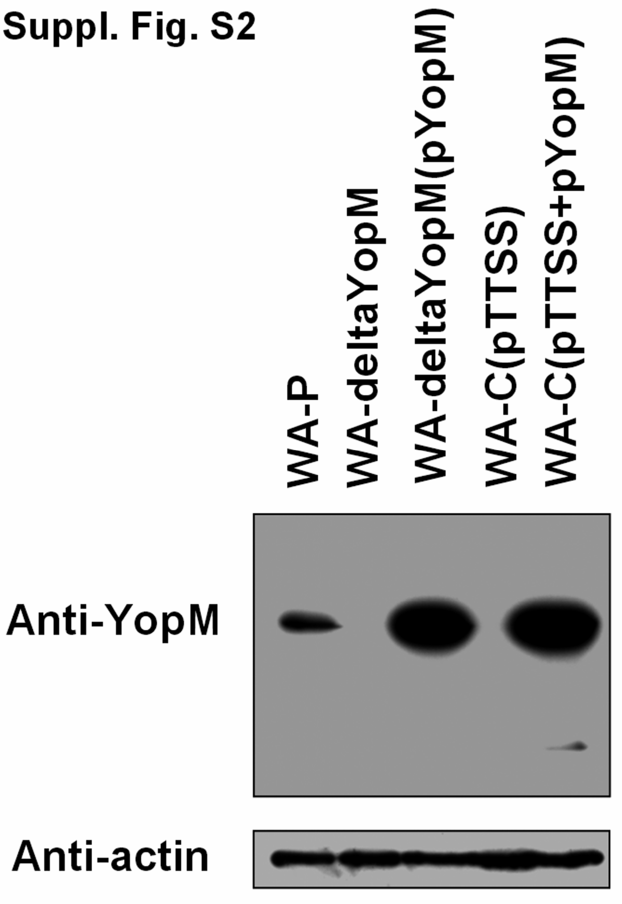

Supplementary Fig. S2: Analysis of YopM quantities translocated into J774.1 cells by different *Yersinia* strains. J774.1 cell were infected with WA-P, WA-deltaYopM, WA-deltaYopM(pYopM), WA-C(pTTSS) and WA-C(pTTSS+YopM) and harvested after 90 min. Cellular lysates were then subjected to western blotting and probed with the indicated antibodies.
